# Cryo-EM structure of the nucleosome containing the *ALB1* enhancer DNA sequence

**DOI:** 10.1098/rsob.170255

**Published:** 2018-03-21

**Authors:** Yoshimasa Takizawa, Hiroki Tanaka, Shinichi Machida, Masako Koyama, Kazumitsu Maehara, Yasuyuki Ohkawa, Paul A. Wade, Matthias Wolf, Hitoshi Kurumizaka

**Affiliations:** 1 Molecular Cryo-Electron Microscopy Unit, Okinawa Institute of Science and Technology Graduate University, 1919-1 Tancha, Onna-son, Kunigami, Okinawa 904-0495, Japan; 2 Laboratory of Structural Biology, Graduate School of Advanced Science and Engineering, Waseda University, 2-2 Wakamatsu-cho, Shinjuku-ku, Tokyo 162-8480, Japan; 3 Institute for Medical-oriented Structural Biology, Waseda University, 2-2 Wakamatsu-cho, Shinjuku-ku, Tokyo 162-8480, Japan; 4 Division of Transcriptomics, Medical Institute of Bioregulation, Kyushu University, 3-1-1 Maidashi, Higashi, Fukuoka 812-0054, Japan; 5 Epigenetics and Stem Cell Biology Laboratory, National Institute of Environmental Health Sciences, Research Triangle Park, NC, USA

**Keywords:** FoxA, histone, nucleosome binding, nucleosome positioning, pioneer transcription factor

## Abstract

Pioneer transcription factors specifically target their recognition DNA sequences within nucleosomes. FoxA is the pioneer transcription factor that binds to the *ALB1* gene enhancer in liver precursor cells, and is required for liver differentiation in embryos. The *ALB1* enhancer DNA sequence is reportedly incorporated into nucleosomes in cells, although the nucleosome structure containing the targeting sites for FoxA has not been clarified yet. In this study, we determined the nucleosome structure containing the *ALB1* enhancer (N1) sequence, by cryogenic electron microscopy at 4.0 Å resolution. The nucleosome structure with the *ALB1* enhancer DNA is not significantly different from the previously reported nucleosome structure with the Widom 601 DNA. Interestingly, in the nucleosomes, the *ALB1* enhancer DNA contains local flexible regions, as compared to the Widom 601 DNA. Consistently, DNaseI treatments revealed that, in the nucleosome, the *ALB1* enhancer (N1) DNA is more accessible than the Widom 601 sequence. The histones also associated less strongly with the *ALB1* enhancer (N1) DNA than the Widom 601 DNA in the nucleosome. Therefore, the local histone–DNA contacts may be responsible for the enhanced DNA accessibility in the nucleosome with the *ALB1* enhancer DNA.

## 1. Introduction

In eukaryotes, genomic DNA is organized into chromatin, in which the nucleosome is the fundamental unit [1]. The protein components of the nucleosome are two histone H2A–H2B dimers and two histone H3–H4 dimers, which associate as a histone octamer [2]. A DNA segment of about 145 base pairs is then wrapped around the histone octamer in the nucleosome [2]. In chromatin, nucleosomes are connected by linker DNAs, and linker histones bind to the DNA region lying on the dyad axis of the nucleosome together with the linker DNAs [1,3,4].

Nucleosome formation generally restricts the binding of transcription factors (TFs) to the DNA wrapped around the histone octamer. Therefore, in active promoters, TFs predominantly bind nucleosome-free DNA regions [5]. By contrast, pioneer transcription factors bind their target sequences within nucleosomes, and create open chromatin conformations around their target sequences [6–8]. This nucleosome reconfiguration by pioneer TFs may be an initial step in the gene activation cascades required for tissue development [9–11].

FoxA (FoxA1, FoxA2 and FoxA3) is a pioneer TF [6–8]. FoxA binds nucleosomal target sequences at the *ALB1* enhancer locus [12,13]. Interestingly, in the nucleosome containing the *ALB1* enhancer (N1) sequence, the FoxA target sequence is located near the nucleosomal dyad axis, which overlaps the linker histone-binding site [3,4], and FoxA competes with the linker histone H1 for nucleosome binding [12,14]. This FoxA-mediated linker histone removal may generate a more relaxed nucleosome configuration [14,15].

In order to understand how FoxA functions as a pioneer TF, it is essential to determine the three-dimensional structure and physical characteristics of its target nucleosome. However, the nucleosome structure containing a native DNA sequence with pioneer TF-binding sites has not been reported yet. In the present study, we reconstituted the nucleosome containing the specific *ALB1* enhancer (N1) sequence, and reconstructed the *ALB1* nucleosome structure by cryogenic electron microscopy to near-atomic resolution.

## 2. Results

### 2.1. Nucleosome reconstitution with the *ALB1* enhancer (N1) DNA

**Figure 1.**
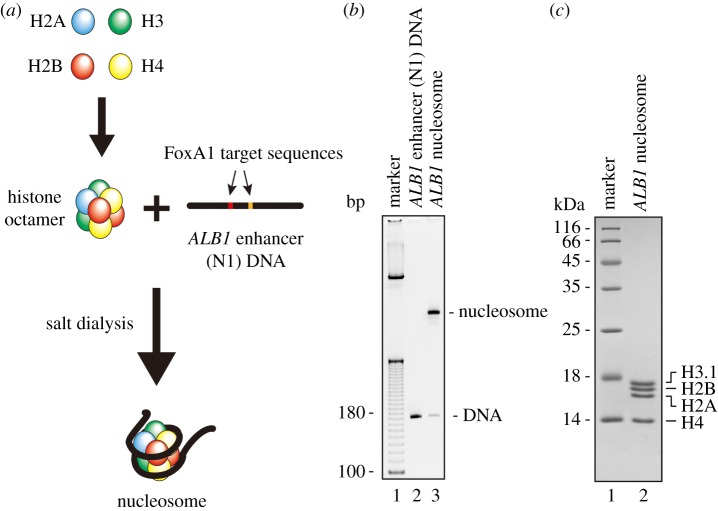
Preparation of the nucleosome with the *ALB1* enhancer (N1) DNA. (*a*) Schematic representation of the nucleosome reconstitution. The histone octamer was reconstituted with the four human core histones (H2A, H2B, H3.1 and H4). The mouse *ALB1* enhancer (N1) DNA was then mixed with the human histone octamer, and the *ALB1* nucleosome was reconstituted by the salt dialysis method. The two FoxA1-binding sequences in the *ALB1* enhancer, eG and eH, are highlighted. (*b*) The purified *ALB1* nucleosome was analysed by non-denaturing 6% PAGE with ethidium bromide staining. (*c*) The purified *ALB1* nucleosome was analysed by 18% SDS-PAGE with Coomassie brilliant blue staining.

A genome-wide DNA sequence analysis revealed that the *ALB1* enhancer (N1) sequence is incorporated into nucleosomes in cells [14,16]. Therefore, we reconstituted the nucleosome with the mouse *ALB1* enhancer (N1) sequence (180 base pairs) [12]. To do so, the histone octamer was formed with human recombinant histones H2A, H2B, H3.1 and H4, and the nucleosome was reconstituted by the salt-dialysis method (figure 1*a*). The reconstituted *ALB1* nucleosome was purified by native PAGE (figure 1*b*). The purified *ALB1* nucleosome contained stoichiometric amounts of human histones H2A, H2B, H3.1 and H4, indicating that the nucleosome was properly formed (figure 1*c*).

### 2.2. Nucleosome positioning on the *ALB1* enhancer (N1) DNA

**Figure 2.**
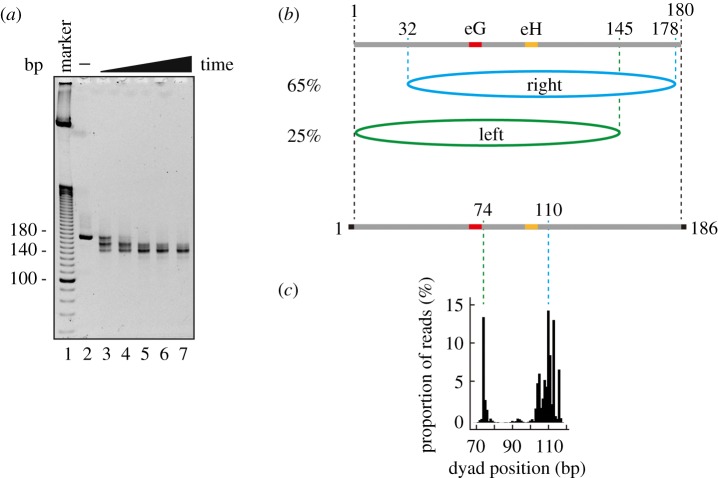
Translational positions of the *ALB1* nucleosome. (*a*) MNase treatment of the *ALB1* nucleosome. The *ALB1* nucleosome containing the 180 base-pair *ALB1* enhancer (N1) DNA was cross-linked by formaldehyde, and then treated with MNase for 0, 2, 4, 8, 12 and 20 min at 37°C. The reactions were stopped by adding a proteinase K solution, containing SDS and EDTA, and the products were analysed by non-denaturing 8% PAGE with ethidium bromide staining. (*b*) The DNA fragments (about 145 base pairs), generated by the MNase treatment of the nucleosome containing the 180 base-pair *ALB1* enhancer (N1) DNA, were inserted into the pGEM-T-Easy vector and sequenced (see Material and methods for details). Twenty DNA fragments were aligned according to their sequences, which showed that 65% of the *ALB1* nucleosomes are in the ‘right position’ and 25% are in the ‘left position’. (*c*) The DNA fragments generated by the MNase treatment of the nucleosome containing the 186 base-pair *ALB1* enhancer (N1) DNA were analysed by deep sequencing using an Illumina MiSeq (Illumina K.K.; USA), and the nucleosome dyad positions were plotted as the position shifted by 73 base pairs from the 5′-end of the reads.

To map the nucleosome positioning, the *ALB1* nucleosome reconstituted with the 180 base-pair *ALB1* enhancer (N1) DNA sequence was fixed with 0.5% formaldehyde, and was then treated with MNase, which preferentially digests linker DNA regions. As shown in figure 2*a*, about 145 base-pair DNA fragments of the *ALB1* enhancer (N1) DNA were protected from the MNase attack, suggesting that the protected DNA region is tightly wrapped within the nucleosome. We then sequenced 20 MNase-resistant DNA fragments, and found that 65% of the DNA fragments were mapped to 2 base pairs away from one end of the 180 base-pair DNA (figure 2*b*, right position), although 25% of the MNase-resistant DNA fragments were mapped to the opposite end of the 180 base-pair DNA (figure 2*b*, left position). Therefore, the right position may be the predominant translational nucleosome position in the 180 base-pair *ALB1* enhancer (N1) DNA.

To confirm these nucleosome positions, we reconstituted the nucleosome with the 180 base-pair *ALB1* enhancer (N1) DNA sequence containing three additional base pairs at both ends (the 186 base-pair *ALB1* DNA). We performed the MNase treatment assay without cross-linking, and the sequences of the resulting DNA fragments were analysed by massive parallel sequencing. The centres of the MNase-resistant DNA fragments, which may correspond to the nucleosomal dyad, were plotted (figure 2*c*). Consistent with the MNase mapping data with cross-linking (figure 2*b*), two nucleosome positions, corresponding to the right and left positions, were found on the *ALB1* enhancer (N1) sequence in the non-cross-linked nucleosomes (figure 2*c*).

### 2.3. Cryo-EM structure of the nucleosome containing the *ALB1* enhancer (N1) DNA sequence

**Figure 3.**
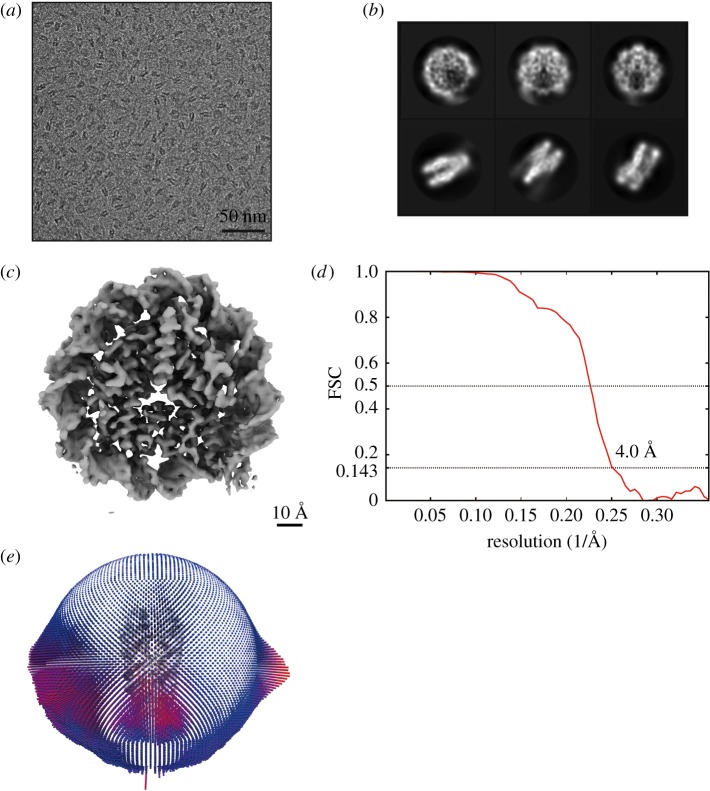
The cryo-EM structure of the *ALB1* nucleosome at 4.0 Å. (*a*) Representative area from a digital cryo-electron micrograph of the *ALB1* nucleosome in amorphous ice. Scale bar indicates 50 nm. (*b*) Selected two-dimensional class averages from single particle images of the *ALB1* nucleosome. Box size is 19.6 nm. (*c*) Cryo-EM iso-potential map of the *ALB1* nucleosome, contoured at 5.3 sigma above mean density. Scale bar indicates 10 Å. (*d*) Gold-standard FSC curve between independently refined reconstructions. The overall resolution of the *ALB1* nucleosome is 4.0 Å at Fourier Shell Correlation (FSC) = 0.143. (*e*) Euler angle distribution of the *ALB1* nucleosome particles contributing to the final reconstruction.

The reconstituted nucleosome with the 186 base-pair *ALB1* enhancer (N1) sequence was fixed with paraformaldehyde by the GraFix method [17]. We then collected images of the *ALB1* nucleosome by the cryo-electron microscopy (cryo-EM) method (figure 3*a,b*). The cryo-EM structures of the *ALB1* nucleosome were reconstructed, and the best three-dimensional class was selected and refined to 4.0 Å resolution (figure 3*c–e* and table 1). In this analysis, we prepared the sample in the presence of FoxA1(170–472), which includes the DNA-binding and histone-binding domains [15]. However, we could not detect the extra volume corresponding to FoxA1(170–472) around the predicted FoxA-binding sites, suggesting that it may have dissociated during the sample preparation process. Although the three-dimensional classification seeks to separate unique three-dimensional structures, the orientation of the DNA sequence cannot be clearly distinguished in the original nucleosome images. We then symmetrized the images. In addition, our cryo-EM nucleosome structure may represent a mixture of two differently positioned nucleosomes (figure 2). Therefore, the DNA may appear as an average structure. In the structure, the nucleosomal DNA corresponding to 146 base pairs was clearly visible, but the 40 base-pair linker DNA segments disappeared, probably due to the symmetry imposition (figure 3*c*).

**Table 1.** Cryo-EM data collection and image processing.

	EMD-6838	EMD-6898
electron microscope	Talos Arctica	Talos Arctica
detector	Falcon 3	Falcon 3
voltage (kV)	200	200
pixel size (Å)	1.40	1.40
exposure time (s)	2	2
movie frames (no.)	79	79
electron dose (e–Å^−2^)	∼80	∼80
defocus range (μm)	−1.5 to −3.0	−1.5 to −3.0
software	RELION 2.1	RELION 2.1
final particles (no.)	236 386	139 343
symmetry	C2	C1
precision of rotations (°)	2.496	2.766
precision of translations (pix)	0.752	0.817
B-factor (Å^2^)	−325	−331
final resolution (Å)	4.0	4.5
FSC criterion	0.143	0.143

### 2.4. Cryo-EM structure of the *ALB1* nucleosome with a linker DNA

**Figure 4.**
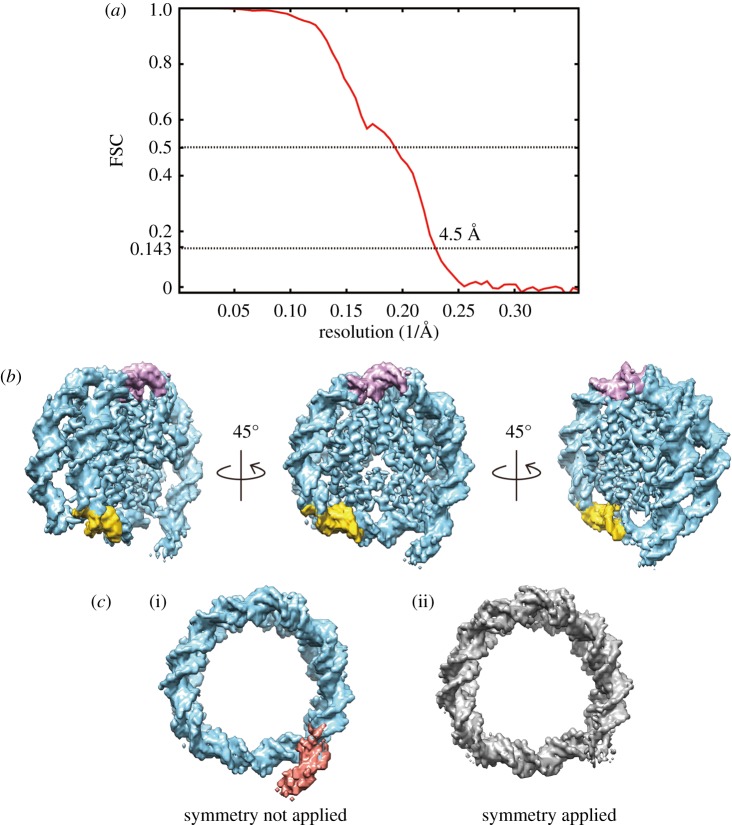
The structure of the *ALB1* nucleosome with linker DNA. (*a*) Gold-standard FSC curve between independently refined reconstructions without symmetry imposition. The overall resolution of the *ALB1* nucleosome with the linker DNA is 4.5 Å at FSC = 0.143. (*b*) Cryo-EM iso-potential map of the *ALB1* nucleosome, contoured at 5.3 sigma above mean density. Three views are presented. The locations of the FoxA1 target DNA sites, eG and eH, are coloured pink and yellow, respectively. (*c*) The DNA moieties of the *ALB1* nucleosome structures. The linker DNA is visible in the structure reconstructed without symmetry imposition ((i); coloured red), but not in the symmetry-applied structure (ii).

To visualize the linker DNA region, we next reconstructed the cryo-EM structure of the *ALB1* nucleosome without the symmetrizing process. The *ALB1* nucleosome was then successfully reconstructed at 4.5 Å resolution (figure 4*a,b*). This time, the linker DNA region was clearly visualized, extending the reconstructed electron potential of the DNA ends beyond the limits of the symmetrized reconstruction (figure 4*b,c*). As shown in figure 2, the right position is the major position of the *ALB1* nucleosome. Therefore, we superimposed the *ALB1* enhancer DNA sequence on the structure by orienting the linker sequence towards the protruding DNA end, and thus mapped the possible locations of the two FoxA-binding sites on the nucleosome structure (figure 4*b*). We used the *ALB1* nucleosome structure for further comparisons with the known nucleosome structure.

### 2.5. The *ALB1* enhancer (N1) DNA structure in the nucleosome

**Figure 5.**
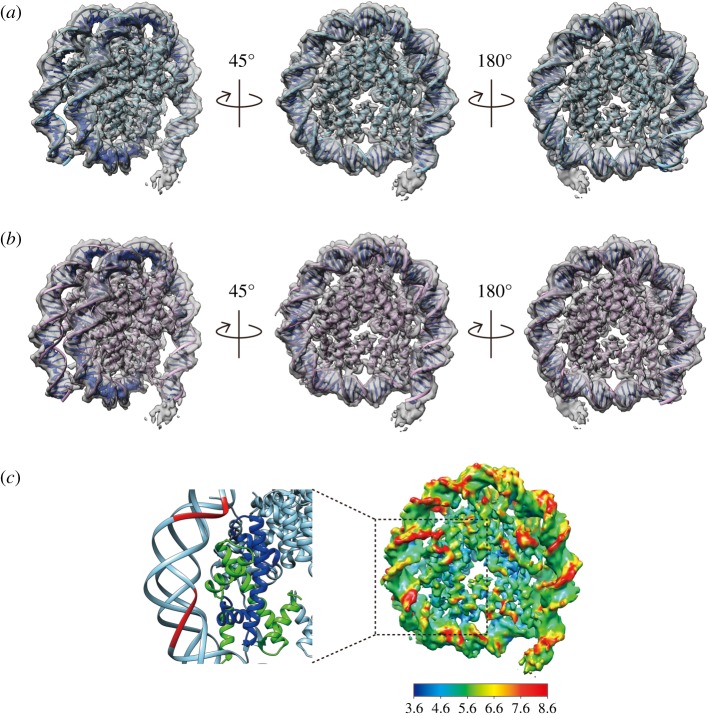
Comparison of the *ALB1* nucleosome, the Widom 601 nucleosome and the MMTV nucleosome. (*a*) Various views of the superimposition of the cryo-EM map (surface rendered in grey) of the *ALB1* nucleosome structure, contoured at 5.3 sigma above mean density, and the crystal structure of the Widom 601 nucleosome (PDB: 3LZ0). The side views of the left and right panels are related by 45° rotation and 180° rotation to the disc view (centre panel) on the vertical axis, respectively. (*b*) The superimposition of the cryo-EM map (surface rendered in grey) of the *ALB1* nucleosome structure, contoured at 5.3 sigma above mean density, and the crystal structure of the MMTV nucleosome (PDB: 5F99). (*c*) Local resolution map of the *ALB1* nucleosome, showing the resolution range across the map from 3.6 Å to 8.6 Å (right panel). A close-up view of the Widom 601 nucleosome region (crystal structure) corresponding to the *ALB1* nucleosome region (dashed line box) is shown in the left panel. Histones H3 and H4 are coloured green and blue, respectively. The low-resolution (lower than 8.6 Å) DNA region of the cryo-EM *ALB1* nucleosome is mapped on the Widom 601 nucleosome structure and coloured red.

We then compared the *ALB1* enhancer (N1) DNA structure with the Widom 601 DNA structure in the nucleosomes. The Widom 601 sequence is a well-known nucleosome positioning sequence that is tightly wrapped around the histone octamer [18,19]. A previous cryo-EM analysis revealed that, up to a resolution of 3.9 Å, the Widom 601 DNA structure in the nucleosome is identical to that in the crystal structure [20]. Therefore, we superimposed the crystal structure of the nucleosomal Widom 601 DNA onto the *ALB1* enhancer (N1) DNA structure. As shown in figure 5*a*, the DNA path in the *ALB1* nucleosome is quite similar to that in the Widom 601 nucleosome, and no obvious difference is apparent. The DNA path of the *ALB1* nucleosome is also similar to that in the nucleosome containing the 3′-LTR of the mouse mammary tumour virus sequence (figure 5*b*) [21]. The local resolution map revealed that the *ALB1* enhancer (N1) DNA contained DNA regions with low resolution, suggesting that these DNA regions are flexible in the nucleosome (figure 5*c* (right panel), coloured red). By contrast, these flexible regions are not obvious in the local resolution map of the cryo-EM Widom 601 nucleosome structure [20]. Interestingly, the flexible DNA regions of the *ALB1* nucleosome are located near the direct binding sites for histones (figure 5*c* (left panel)). This fact suggested that when compared with the Widom 601 DNA, the *ALB1* enhancer (N1) DNA may be loosely bound to the histones in the nucleosome.

### 2.6. The nucleosome containing the *ALB1* enhancer (N1) DNA is more accessible to DNaseI than that containing the Widom 601 DNA

**Figure 6.**
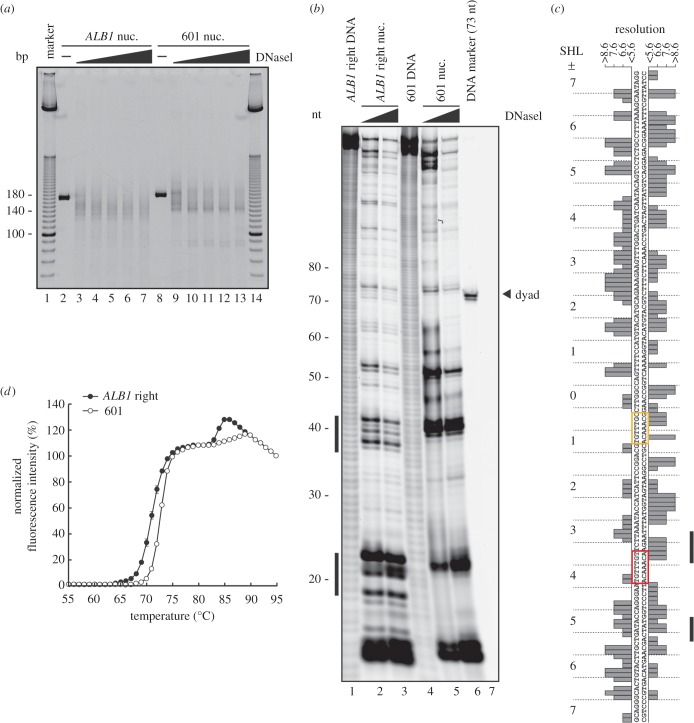
DNA accessibility and nucleosome stability. (*a*) DNaseI treatment assay. The nucleosomes containing the 186 base-pair *ALB1* enhancer DNA and the 193 base-pair Widom 601 DNA were treated with DNaseI. The reactions were stopped by adding a proteinase K solution, containing SDS and EDTA, and the products were analysed by non-denaturing 8% PAGE with ethidium bromide staining. (*b*) DNaseI footprinting assay. The nucleosomes containing 147 base pairs of the *ALB1* enhancer DNA (right position) or the Widom 601 DNA were treated with DNaseI (1 and 4 units). The reactions were stopped by adding a proteinase K solution containing EDTA. The purified DNA products were analysed by denaturing 8% PAGE, and were detected by the 5′ end-labelled Cy5 fluorescence. (*c*) The local resolution of the *ALB1* nucleosome structure, plotted against the DNA sequence of the *ALB1* nucleosome. Two columns of bars indicate two strands of DNA on the ALB1 nucleosome. The DNA sequence is aligned according to the right nucleosome position, and the superhelical locations (SHLs) of the nucleosomes are presented. The DNA sequences of the FoxA1 target DNA sites, eG and eH, are surrounded by red and yellow boxes, respectively. The DNaseI hypersensitive sites shown in (*b*) are presented on the right side of the figure. The graph bars correspond to the local resolutions of the *ALB1* nucleosome cryo-EM reconstruction, as shown in figure 5*c*. (*d*) Thermal stability curves of the *ALB1* nucleosome (147 base pairs, right position) and the Widom 601 nucleosome (147 base pairs). The normalized fluorescence intensities of the *ALB1* and Widom 601 nucleosome samples were plotted at each temperature, from 55°C to 95°C. Standard deviation values are shown (*n* = 3).

To test whether the *ALB1*enhancer (N1) DNA is actually loosened, we performed a DNaseI treatment assay. In this assay, the endonuclease DNaseI attacks more efficiently if the nucleosomal DNA is loosened. We found that the nucleosomal *ALB1* enhancer (N1) DNA (186 base pairs) was more susceptible to the DNaseI than the nucleosomal Widom 601 DNA (figure 6*a*). We then identified the DNaseI-sensitive sites of the *ALB1* enhancer (N1) DNA in the nucleosome, by denaturing polyacrylamide gel electrophoresis. In the *ALB1* nucleosome with the right position, the DNA regions around 20 bases and 40 bases from the labelled end were DNaseI-sensitive sites (figure 6*b*). These regions coincide with the low-resolution DNA regions of the *ALB1* nucleosome structure (figure 6*c*). Therefore, we conclude that the *ALB1* enhancer (N1) DNA is locally loosened in the nucleosome.

We then compared the thermal stabilities of the *ALB1* nucleosome and the Widom 601 nucleosome. To eliminate the effect of the nucleosome positions, we reconstituted the *ALB1* (right position) and Widom 601 nucleosomes with 147 base-pair DNAs. In the thermal stability assay, the dissociations of the H2A–H2B and H3–H4 dimers from the nucleosome are independently monitored, as the first and second peaks, respectively. In the Widom 601 nucleosome, the H2A–H2B dimers dissociated at 70–75°C, while the second peak for the H3–H4 dimer dissociation was observed at 86–90°C (figure 6*d*). Interestingly, in the *ALB1* nucleosome, the first and second peaks were both shifted towards lower temperatures (figure 6*d*). These data indicate that the histones of the *ALB1* nucleosome associate more weakly with the DNA than those of the Widom 601 nucleosome, consistent with our structural analysis. Therefore, the *ALB1* enhancer (N1) DNA sequence may be more accessible to DNA-binding proteins, because of the loosened association of the DNA with the histones. This characteristic of the nucleosomal *ALB1* enhancer DNA may play a role in accommodating the pioneer TFs within the nucleosome.

## 3. Discussion

The nucleosomal DNA binding of the proteins that regulate genomic DNA functions, such as replication, recombination, repair and transcription, depends on the sequence-dependent conformations and physical properties of the DNA wrapped in nucleosomes, as well as their histone compositions. As a consequence, structural studies of nucleosomal DNA by X-ray crystallography have been severely limited, because the crystallization of the nucleosome is highly contingent on the DNA sequence. In fact, many crystal structures of nucleosomes have been deposited in the Protein Data Bank, but most of them contained the palindromic α-satellite or Widom 601 DNA sequence with 145–147 base pairs [2,19,22–24]. These DNA sequences form relatively stable nucleosomes, which are properly packed in the crystal. However, to understand the mechanism by which the genomic DNA is regulated in chromatin, the structures and physical properties of nucleosomes containing native genome sequences must be studied. In this context, a crystal structure of the nucleosome containing the 3′-LTR of the mouse mammary tumour virus sequence has been reported [21]. This is the only nucleosome structure containing a native regulatory DNA sequence published so far.

Currently, the cryo-EM method is becoming increasingly used for structural studies of nucleosomes. A high-resolution cryo-EM nucleosome structure has been reported at 3.9 Å resolution [20]. The cryo-EM structure of the nucleosome complexed with a retroviral integrase has been determined at 7.8 Å resolution [25]. The nucleosome with H2A lysine 15 (H2AK15) monoubiquitination and H4 lysine 20 (H4K20) methylation was reconstructed in a complex form with 53BP1, which functions in the double-strand break repair process, by cryo-EM at 4.5 Å resolution [26]. The nucleosome structure complexed with the domains of a nucleosome remodeller, yeast Chd1, has also been reconstructed at 4.8 Å resolution [27]. In addition to these mononucleosome structures, a cryo-EM structure of the polynucleosome containing a linker histone H1 has been determined at 11 Å resolution [28]. These outstanding studies have greatly advanced the structural biology of chromatin. However, these cryo-EM structures of nucleosomes were reconstituted with the Widom 601 DNA sequence, except for the nucleosome complexed with a retroviral integrase [25].

In this study, we successfully reconstructed the nucleosome structure with 186 base pairs of DNA containing the *ALB1* enhancer (N1) sequence by the cryo-EM method at near-atomic resolution. This method allows us to analyse the nucleosome structure with a native DNA sequence. In addition, the cryo-EM method can avoid the potential restriction of the DNA structure by the effects of crystal packing.

We found that the *ALB1* enhancer (N1) DNA exhibited higher accessibility to DNaseI than the Widom 601 sequence in the nucleosome (figure 6), although the DNA-binding paths of both nucleosomes are not significantly different (figures 3–5)*.* This higher accessibility suggests that the *ALB1* enhancer (N1) DNA in the nucleosome may allow the efficient binding of pioneer TFs, such as FoxA, to the nucleosomal DNA. Interestingly, in the nucleosome, the *ALB1* enhancer (N1) sequence contains flexible regions, and the DNA regions are located near a putative FoxA-binding region (figures 4 and 6). We also found that the histones are more weakly associated in the *ALB1* nucleosome than in the Widom 601 nucleosome (figure 6). Therefore, the enhanced DNA accessibility and the weaker histone association found in the *ALB1* nucleosome may be induced by the reduced local histone–DNA contacts. Unfortunately, the resolution of our cryo-EM structures (4.0–4.5 Å) is not high enough to clarify the detailed histone–DNA interactions in the *ALB1* nucleosome. Further structural studies will be required to reveal the mechanism by which the association of the histones is weakened and how the *ALB1* enhancer (N1) DNA sequence becomes accessible to pioneer TFs in the nucleosome.

## 4. Material and methods

### 4.1. Purification of recombinant histones

All human histones were prepared as recombinant proteins, and were purified by the method described previously [29,30].

### 4.2. Purification of nucleosomes

The 186 base-pair mouse *ALB1* enhancer (N1) DNA fragment [12,16,31] (sequence: ATCCGAGATGGTACTTTGTGTCTCCTGCTCTGTCAGCAGGGCACTGTACTTGCTGATACCAGGGAATGTTTGTTCTTAAATACCATCATTCCGGACGTGTTTGCCTTGGCCAGTTTTCCATGTACATGCAGAAAGAAGTTTGGACTGATCAATACAGTCCTCTGCCTTTAAAGCAATAGGAAAGAT) and the 193 base-pair DNA fragment containing the Widom 601 sequence [18,32] (sequence: ATCGGACCCTATCGCGAGCCAGGCCTGAGAATCCGGTGCCGAGGCCGCTCAATTGGTCGTAGACAGCTCTAGCACCGCTTAAACGCACGTACGCGCTGTCCCCCGCGTTTTAACCGCCAAGGGGATTACTCCCTAGTCTCCAGGCACGTGTCAGATATATACATCCAGGCCTTGTGTCGCGAAATTCATAGAT) were purified by the methods described previously [33]. The 180 base-pair mouse *ALB1* enhancer DNA fragment, which was missing three bases at both ends (derived from the *Eco*RV site) of the 186 base-pair *ALB1* DNA, was amplified by PCR and purified by non-denaturing 6% PAGE, using a Prep Cell apparatus (Bio-Rad). For the reconstitution of the histone octamer, human H2A, H2B, H3.1 and H4 were mixed in denaturing buffer (20 mM Tris–HCl (pH 7.5), 7 M guanidine hydrochloride and 20 mM 2-mercaptoethanol), and the mixture was rotated at 4°C for 1.5 h, followed by dialysis against refolding buffer (10 mM Tris–HCl (pH 7.5), 2 M NaCl, 1 mM EDTA, and 5 mM 2-mercaptoethanol). The resulting human histone octamer was further purified by Superdex200 (GE Healthcare) gel filtration chromatography. The *ALB1* nucleosome and the 601 nucleosome were reconstituted with the histone octamer and the *ALB1* DNA fragment and the Widom 601 DNA fragment, respectively, by the salt dialysis method [29,34]. The reconstituted nucleosomes were finally purified by non-denaturing 6% PAGE, using a Prep Cell apparatus (Bio-Rad). The nucleosomes were collected in TCS buffer (20 mM Tris–HCl (pH 7.5) and 1 mM DTT) and were concentrated using a Millipore concentrator (MW cut-off 30 000).

### 4.3. Mapping of nucleosome positioning by micrococcal nuclease treatment

The nucleosome containing the 180 base-pair *ALB1* enhancer DNA was dialysed against 20 mM HEPES-NaOH (pH 7.5) buffer containing 1 mM DTT, and was treated with 0.5% formaldehyde at 25°C for 30 s. The reaction was stopped by adding glycine to a final concentration of 200 mM, and the sample was dialysed against TCS buffer. The cross-linked *ALB1* nucleosome (0.6 µg of DNA) was incubated at 37°C for the indicated times in the presence of 0.24 units of micrococcal nuclease (MNase; Takara), in 60 µl of reaction solution (50 mM Tris–HCl (pH 8.0), 25 mM NaCl, 2.5 mM CaCl_2_ and 1.9 mM DTT). After the incubation, each reaction aliquot (10 µl) was stopped by adding 5 µl of deproteinization solution (0.25 mg ml^−1^ proteinase K solution (Roche), 20 mM Tris–HCl (pH 8.0), 20 mM EDTA, and 0.1% SDS). For decross-linking, NaCl was added to the sample to a final concentration of 420 mM, and the sample was incubated overnight at 65°C. The samples were then analysed by non-denaturing 8% PAGE in 0.5× TBE buffer. The DNA bands were visualized by ethidium bromide staining. The resulting DNA fragments (about 145 base pairs) were purified by the Wizard® SV Gel and PCR Clean-Up System (Promega). The DNA ends were treated with Klenow polymerase, and the 5′-ends were phosphorylated by T4 polynucleotide kinase. The products were ligated into the pGEM-T-Easy vector, which was digested and dephosphorylated by *Eco*RV and calf intestinal alkaline phosphatase (CIAP), respectively, prior to insert ligation. The sequences of the inserted DNA fragments were analysed by using primers with sequences complementary to those flanking the insert DNA.

### 4.4. Deep sequencing analysis of the nucleosome positioning

Purified nucleosomes, containing the 186 base-pair *ALB1* enhancer DNA fragment, were treated with MNase (0.5 units µg^−1^ DNA). The resulting DNA fragments (about 145 base pairs) were purified by the Wizard® SV Gel and PCR Clean-Up System (Promega). The purified DNA fragments were then sequenced, using an Illumina MiSeq (Illumina K.K.; USA). To reconstruct the full-length nucleosomal DNAs from the paired-end reads of the MNase-seq, each pair was transformed (concatenated) into the single-end read using FLASH [35] (version: 1.2.11, options: -m 10 -M 200). We restricted the reads to lengths of 147 ± 10 bp, to eliminate the non-nucleosomal DNA fragments. The concatenated reads were mapped to the N1 sequence using Bowtie [36] (version: 1.2, options: -v3 -m1). The distribution of dyad positions was estimated as the proportions (count/total) of mid-points of the mapped reads at each 1 bp position in the N1 sequence.

### 4.5. Grafix

Nucleosomes (4 µM) containing the 186 base-pair *ALB1* enhancer DNA fragment were mixed with FoxA1(170–472) (12.2 µM), in 0.1 ml of reaction buffer containing 20 mM Tris–HCl (pH 7.5), 50 mM NaCl, 0.25 mM MgCl_2_, 0.25 mM 2-mercaptoethanol and 0.25 mM DTT. The reaction mixture was incubated for 30 min at room temperature. After the incubation, the sample was applied onto the top of the gradient solution (5–25% sucrose gradient with 0–4% paraformaldehyde, in 10 mM HEPES-NaOH (pH 7.5) buffer containing 20 mM NaCl and 1 mM DTT), and was centrifuged at 27 000 r.p.m. in an SW28 rotor (Beckman Coulter) at 4°C for 16 h. After the ultracentrifugation, 1.3 ml fractions were collected from the top of the gradient. The absorbance (260 nm) was monitored for each fraction, and the peak fractions were dialysed three times against 10 mM HEPES buffer (pH 7.5) containing 1 mM DTT.

### 4.6. Cryo-electron microscopy

Aliquots (2.5 µl) of the purified *ALB1* nucleosome mixed with the human FoxA1 deletion mutant, FoxA1(170–472), which contains both the DNA-binding and histone-binding domains, were applied to Quantifoil holey carbon grids (R1.2/1.3 200-mesh Cu), which were freshly cleaned using a Solarus Plasma Cleaner (Gatan, Pleasanton, USA) for 15 s at 20 W in a 23% H_2_, 77% O_2_ gas mix. The grids were blotted for 3 s at 16°C and 100% relative humidity, and then immediate plunge-frozen in liquid ethane with a Vitrobot Mark IV (Thermo Fisher, Hillsboro, USA). Cryo-EM data were collected using the EPU automation software on a Talos Arctica microscope (Thermo Fisher, Hillsboro, USA), operating at 200 kV at a calibrated magnification of 100 000× (pixel size of 1.40 Å), with defocus ranging from −1.5 to −3.0 µm. Digital micrographs were recorded with 2-second exposure times on a Falcon 3 direct electron detector (Thermo Fisher, Hillsboro, USA) in the linear mode, at a dose rate of approximately 40 electrons per Å^2^ per second with 25 ms per frame time, retaining a total of 79 frames with an accumulated total dose of approximately 80 electrons per Å^2^.

### 4.7. Image processing

All frames in 2312 movies were aligned using MOTIONCOR2 [37], with dose weighting. The contrast transfer function (CTF) was estimated by CTFFIND4 [38] from digital micrographs, without dose weighting. In total, 1116 micrographs were selected based on good CTF fit correlation to approximately 8 Å resolution with minimal astigmatism. RELION 2.1 [39] was used for all subsequent image processing operations. A total of 1 182 985 particles of the *ALB1* nucleosome were picked semi-automatically with a box-size of 140 × 140 pixels, followed by two rounds of two-dimensional classification to discard bad particles, resulting in the selection of 626 544 particles. The crystal structure of a canonical nucleosome (PDB: 3LZ0), low-pass filtered to 60 Å, was used as an initial alignment model. After the first round of three-dimensional classification, 288 789 particles were selected for three-dimensional refinement, and C2 symmetry was applied to the three-dimensional reconstruction. Based on CTF fit correlation to approximately 5 Å resolution, 236 386 particles were further selected before three-dimensional refinement. The final C2-symmetrized map was sharpened with an exponential B-factor (−325 Å^2^). For the three-dimensional reconstruction without symmetry, a second round of three-dimensional classification was performed using a subset of 288 789 particles, and the reconstruction with the linker DNA containing 139 343 particles was selected for three-dimensional refinement. To sharpen the final unsymmetrized map, an exponential B-factor (−331 Å^2^) was applied. The resolution of each final three-dimensional map was estimated following the gold standard Fourier Shell Correlation (FSC) at FSC = 0.143 [39]. Maps were normalized with MAPMAN [40]. The model of 3LZ0.pdb was docked into the electron potential map of the *ALB1* nucleosome reconstruction with UCSF Chimera [41]. The local resolution map of the *ALB1* nucleosome was created by RESMAP [42]. Iso-electron potential surfaces were visualized with UCSF ChimeraX [43] using the ambient occlusion shader (figure 3*c*) and UCSF Chimera [41] (figures 4 and 5).

### 4.8. DNaseI treatment assay

The nucleosome containing the 186 base-pair *ALB1* enhancer DNA fragment or the 193 base-pair Widom 601 DNA fragment (200 ng of DNA) was incubated with DNaseI (0.01, 0.02, 0.03, 0.04 and 0.05 units) in 10 µl of 35 mM Tris–HCl (pH 8.0) buffer, containing 10 mM NaCl, 1.5 mM MnCl_2_ and 1.6 mM DTT, at 26°C for 15 min. After the incubation, the reactions were stopped by adding 5 µl of deproteinization solution (0.25 mg ml^−1^ proteinase K solution (Roche), 20 mM Tris–HCl (pH 8.0), 20 mM EDTA and 0.1% SDS). The reaction products were analysed by non-denaturing 8% PAGE in 0.5× TBE buffer. The DNA fragments were stained with ethidium bromide.

### 4.9. DNaseI footprinting

For the DNaseI footprinting reaction, purified nucleosomes containing the 5′-Cy5 labelled *ALB1* DNA fragment (right position, 147 base pairs) or a Widom 601 DNA fragment (147 base pairs, 3 µg of DNA) were incubated with DNaseI (1 and 4 units), in 22 µl of reaction buffer (15 mM Tris–HCl (pH 7.5) and 1.5 mM MgCl_2_) at 25°C for 5 min. After the incubation, the reactions were stopped by adding 6 µl of deproteinization solution (33.3 mM EDTA and 12.4 mg ml^−1^ proteinase K solution (Roche)). The DNA fragments were purified by phenol–chloroform extraction and ethanol precipitation. The purified DNA fragments were analysed by denaturing 8% PAGE. The Cy5 fluorescence signal was detected with an Amersham Typhoon scanner (GE Healthcare). The DNA sequences used in DNaseI footprinting are described below.

*ALB1* right position (147 base pairs): AGCAGGGCACTGTACTTGCTGATACCAGGGAATGTTTGTTCTTAAATACCATCATTCCGGACGTGTTTGCCTTGGCCAGTTTTCCATGTACATGCAGAAAGAAGTTTGGACTGATCAATACAGTCCTCTGCCTTTAAAGCAATAGGA

Widom 601 (147 base pairs): ATCGAGAATCCCGGTGCCGAGGCCGCTCAATTGGTCGTAGACAGCTCTAGCACCGCTTAAACGCACGTACGCGCTGTCCCCCGCGTTTTAACCGCCAAGGGGATTACTCCCTAGTCTCCAGGCACGTGTCAGATATATACATCCGAT

### 4.10. Thermal stability assay of nucleosomes

The thermal stability assay was performed by the method described previously [44,45]. Purified nucleosomes containing either the *ALB1* enhancer DNA fragment or the Widom 601 DNA fragment (1.1 µM) were mixed with SYPRO Orange dye (Sigma-Aldrich) in 20 mM Tris–HCl buffer (pH 7.5), containing 100 mM NaCl and 1 mM DTT. The SYPRO Orange fluorescence was monitored with a StepOnePlus™ Real-Time PCR system (Applied Biosystems), using a temperature gradient from 25°C to 95°C, in steps of 1°C min^−1^. The DNA sequences used in the thermal stability assay are described below.

*ALB1* right position (147 base pairs): ATCAGGGCACTGTACTTGCTGATACCAGGGAATGTTTGTTCTTAAATACCATCATTCCGGACGTGTTTGCCTTGGCCAGTTTTCCATGTACATGCAGAAAGAAGTTTGGACTGATCAATACAGTCCTCTGCCTTTAAAGCAATAGAT

Widom 601 (147 base pairs): ATCGAGAATCCCGGTGCCGAGGCCGCTCAATTGGTCGTAGACAGCTCTAGCACCGCTTAAACGCACGTACGCGCTGTCCCCCGCGTTTTAACCGCCAAGGGGATTACTCCCTAGTCTCCAGGCACGTGTCAGATATATACATCCGAT

## Data accessibility

EMDB IDs: The cryo-EM maps of the *ALB1* nucleosomes have been deposited in the Electron Microscopy Data Bank, with the EMDB ID codes EMD-6838 and EMD-6898.
